# Local ecological knowledge concerning the invasion of Amerindian lands in the northern Brazilian Amazon by *Acacia mangium* (Willd.)

**DOI:** 10.1186/s13002-018-0231-x

**Published:** 2018-05-03

**Authors:** Arlene Oliveira Souza, Maria do Perpétuo Socorro Rodrigues Chaves, Reinaldo Imbrozio Barbosa, Charles Roland Clement

**Affiliations:** 1 Programa de Pós-Graduação em Biodiversidade e Biotecnologia da Rede BIONORTE, Av. Carvalho Leal, 1777, Bairro Cachoeirinha, Edifício Anexo, 4° andar, Manaus, AM CEP 69065-001 Brazil; 2 Docente do Curso de Licenciatura em Educação do Campo da Universidade Federal de Roraima, Av. Capitão Ene Garcêz, 2413, Bairro Aeroporto, Campus do Paricarana, Boa Vista, RR CEP 69304-000 Brazil; 3 Pesquisadora do Projeto Nova Cartografia da Amazônia, Núcleo Roraima, Boa Vista, RR Brazil; 4 Universidade Federal do Amazonas, Av. General Rodrigo Octávio, 6200, Coroado I, Manaus, AM CEP 69077-000 Brazil; 5 Instituto Nacional de Pesquisas da Amazônia—INPA/NAPRR, R. Cel. Pinto, 315, Centro, Boa Vista, RR 69301-150 Brazil; 6 Instituto Nacional de Pesquisas da Amazônia—INPA, Avenida André Araújo, 2936, Petrópolis, Manaus, AM 69067-375 Brazil

**Keywords:** Ethnoecology, Traditional knowledge, Biological invasion, Non-native invasive species, Indigenous nations

## Abstract

**Introduction:**

Invasive plants can impact biodiversity as well as the lives of native human populations. Natural ecosystems represent sources of natural resources essential for the subsistence and socio-cultural continuity of these social groups. Approximately 30,000 ha of *Acacia mangium* were planted for commercial purposes in savanna areas surrounding indigenous lands in Roraima State, Brazil, at the end of the 1990s. We examined the local ecological knowledge of indigenous Wapichana and Macuxi Amerindians, members of the *Arawak* and *Carib* linguistic families, respectively, concerning *A. mangium* Willdenow (Fabaceae) in a savanna ecosystem (“*Lavrado*”) to attempt to understand its propagation beyond the limits of the commercial plantations and contribute to mitigating its impacts on socio-ecological systems.

**Methods:**

The present study was undertaken in the Moskow, São Domingos, and Malacacheta communities in the Moskow and Malacacheta Indigenous Lands (ILs) in the Serra da Lua region of Roraima State, in the northern Brazilian Amazon region. Interviews were conducted with a total of 94 indigenous individuals of both sexes, with ages between 18 and 76, and low levels of formal schooling, with an average time of permanence in the area of 21 years; some still spoke only their native languages. The interviews focused on their ecological knowledge of the invasive, non-native *A. mangium* and their uses of it.

**Results:**

The informants affirmed that *A. mangium* negatively impacted the local fauna and flora, making their subsistence more difficult and altering their daily routines. Among the problems cited were alterations of water quality (71.3%), negative impacts on crops (60.6%), negative impacts on the equilibrium of the local fauna (52.1%), increased farm labor requirements (41.5%), and restriction of access to indigenous lands (23.4%). There were no significant differences between the opinions of men and women, nor between community leaders and nonleaders. Most of the interviewees (89%) felt that *A. mangium* had no positive importance for the local economy and saw no future prospects of beneficial use.

**Conclusions:**

The Wapichana and Macuxi informants felt that the invasion by *A. mangium* had caused negative effects on the natural environment and on community subsistence in the indigenous lands due to its rapid and unwanted propagation. The similarity between the opinions of men and women and between community leaders and nonleaders demonstrates the existence of knowledge that is well distributed among these communities and transmitted within their communities through social–cultural interactions.

*It grows on our farm plots soon after they were cleared and burned, and with the coming of the rains. Then the trouble starts. We have to pull out the acacia plants to plant manioc. I can see it growing, the leaves start changing, I used to think that it was a curse.*

(an indigenous informant from the São Domingos community).

## Background

Plant invasions of natural ecosystems can seriously threaten native biodiversity and the subsistence of indigenous peoples [1–4]. Invasive plants can cause a number of environmental impacts, such as loss of native species, alterations in the structure and composition of invaded habitats, and their capacity to provide ecosystem goods and services, resulting in fewer available natural resources essential for the survival of local populations [1, 5, 6]. Invasive species, however, are often important for generating local income in many different parts of the world, as well as providing food resources, medicines, and socio-culturally significant items [7–10]. These contradictory views about invasive plants require detailed studies of the Local Ecological Knowledge (LEK) of the human populations coexisting with them in order to comprehend the equilibrium between positive and negative impacts.

Although scientists have investigated the effects of invasive plants in various parts of the world and the phases of the invasion process [11], there is still a need for more detailed information about LEK and how the propagation of invasive plants impacts the well-being of traditional and/or indigenous populations. Berkes [12] defined LEK as: “the accumulated body of knowledge, practices, and beliefs concerning the evolving relationships between humans and their environment and how that information is culturally transmitted through generations.” Using this approach in ecological research can provide useful perspectives for managing biological invasions and invaded ecosystems [2, 13]. As such, the present study sought to analyze indigenous knowledge concerning the invasion by *Acacia mangium* of indigenous lands in Roraima, Brazil, and to determine how to contribute to mitigating the environmental damage caused by these trees.

The spontaneous propagation of *A. mangium* within indigenous lands is considered an injustice imposed on those communities and has been discussed in numerous community meetings. During these discussions, in addition to doubts about how these plants propagated themselves, community members voiced their concerns about future threats to their socio-biodiversity, guarantees of food security, and the physical and cultural continuity of their indigenous lifestyles [12]. In light of these concerns, community members have taken into their own hands the eradication of invasive plants, principally during the preparation of farm plots for planting, when invasive trees are cut and burned (although the trunks are left in the fields, as their removal is quite labor intensive).

The concerns of the indigenous peoples have been ignored by the administrators of the commercial tree farms ever since they were first planted, even though there are records of formal complaints citing impacts to the local environment. Only a few limited initiatives have been carried out by the administrators (such as the removal of easily accessible acacia trees near the communities)—measures that have not solved the problem. We believe that the negative impacts of these non-native trees will only be minimized after evaluating, together with members of these communities, the factors that aid their propagation. These plans would take into consideration the specificity of the social and natural environment and incorporate efficient proactive components (the inclusion of scientific information concerning the ecology of the invasive species, continuous monitoring to control its propagation, and eradication of adult plants). These actions would reduce seed dispersal, while investments were made in scientific studies of their natural enemies for control purposes.

Researchers examining the perceptions of human societies in relation to the impacts of invasive plants have approached the subject from different angles, with some finding that these plants can take on positive roles [2, 8], while others have focused on their negative impacts on local social dynamics [7]. Based on these studies, there appear to be various schools of thought that support the exploitation of invasive species that cause ecological and social-environmental impacts, and it must be recognized that some invasive species actually assume positive roles in the lives and subsistence activities of rural communities due to their utility and the opportunity of generating extra income and promoting cultural continuity [8]. The species *Acacia delbata*, for example, offers valuable contributions to the subsistence of rural populations in Madagascar, who exploit its wood for fuel in their homes, as well as commercially for railway construction; it also provides shade along roads and avenues. Similarly, *Acacia mearnsii* is sold by rural communities in South Africa for its wood and is used in traditional ceremonies and cultural rituals [7]. Although invasive species are occasionally used to fulfill subsistence necessities, the communities that use them are well aware of the environmental and social problems they generate. In Kenya, where invasion by *Prosopis juliflora* is most advanced, few people recognize any benefits from the plant, while most recognize its many problems, including making agricultural activities more difficult, draining water resources, displacing native trees, and creating obstacles to their movement and transportation needs [14].

Numerous studies have reported serious negative impacts on the food security and cultural resources of indigenous populations, as well as restrictions on free access to previously used resources due to the arrival of those non-native plants [2, 7]. Other studies have noted that these plants produce landscape alterations that upset the natural equilibrium of the regional fauna [2, 14] and put local traditional knowledge of regional biodiversity at risk [2].

One example of an invasive plant with both positive and negative attributes is the tree species *Acacia mangium* Willdenow (Fabaceae), a member of the group known as the “Australian acacias,” which are native to Queensland in Northern Australia, Papua New Guinea, and Indonesia [15]. This species demonstrates vigorous and competitive growth, and rapid adaptation to a wide variety of edapho-climatic conditions [16]. This adaptability favors the silviculture of *A. mangium* for commercial exploitation of its wood and related products (such as cellulose, honey, beeswax, tannins, and charcoal); it can also be used to recuperate degraded soils [16, 17]. Initial interest in producing cellulose and lumber in Roraima resulted in the planting of 30,000 ha of *A. mangium* in natural savanna ecosystems (“Lavrado”) during the early 1990s, near the lands of the Wapichana and Macuxi Amerindians. “Lavrado” is the regional designation for the local savanna eco-region [18]. According to Barbosa et al. [18], savanna ecosystems are principally characterized by phytophysiognomies with grassy substrates and low densities of trees and shrubs that do not form a continuous canopy layer. This type of ecosystem supports many animal and plant species essential to native human populations that have been managed based on traditional knowledge, guaranteeing the continuity of these material and cultural resources.

The Wapichana and Macuxi people have had more than two decades of contact with *A. mangium* (often called the “foreign plant”), which has favored the accumulation of local ecological knowledge about this non-native species as it spread through their lands in Roraima State, Brazil [2]. The interactions between this invasive species and other plants in their farm plots have been discussed in community meetings—as shown in the quote that opens the introduction of this text.

The Wapichana and Macuxi have attempted to defend their territory by publicizing the invasions of their farm plots by *A. mangium*, as well as the alterations in the color of the water; the lowering of water levels in rivers, wetlands, and hand-dug wells; losses of crop production; and the exaggerated abundance of highly aggressive Africanized bees *Apis mellifera scutellata*, another invasive species that is adapted to inhospitable environments and demonstrates a higher reproductive capacity and a shorter life cycle than other subspecies in Brazil [19]. According to their local ecological knowledge, the cultivation of *A. mangium* threatens the cultural and material continuity of the Wapichana and Macuxi in the region [2].

Local ecological knowledge concerning the impacts of invasive plants on the survival of traditional populations is a recent focus of research. Siges [9] analyzed the different uses of the invasive species *Piper aduncum* according to the ages of members of rural communities in Africa, as well as the different perceptions of men and women and older and younger people concerning that plant. Older people demonstrated significant concerns about the loss of forest products following the invasion by *P. aduncum*, and men cited the substitution of other food plants by sweet potatoes (which grow well in the presence of the invader). For men, the presence of *P. aduncum* made the plots easier to tend by women, although the women noted the disappearance of mushrooms that were previously encountered in the forest edges. The authors also noted divergence among the informants in terms of the effects of *P. aduncum* on food production: with some residents complaining of losses of tuber production and others stating the exact opposite. Another study focusing on the invasive plant *Piper juliflora* in Kenya indicated that its effects on subsistence production were not significant in terms of local perceptions [14]. While rural communities in Nepal use the invasive species *Mikania micrantha* in subsistence activities, they evaluated it as an ecological evil—suggesting that local perceptions can vary over time according to different intensities of invasion. Local populations observed decreases in the production rates of forest products, but many were not able to accurately evaluate production over the last 5 years and felt that there was actually an increase in resource availability [20]. The rapid dissemination of the invasive species *Lantana camara* can be attributed to its prolific fruit production, the ample dispersal of its seeds, changes in fire management practices, and the traditional harvesting of grass and bamboo; younger members of the local population mentioned that *Lantana* promptly re-sprouts after burning [2].

In light of the fact that indigenous communities in Roraima have experienced severe social-environmental impacts due to the rapid spread of *A. mangium* [3], we undertook an analysis of this invasive species considering the specific natural and social environments of these communities to identify the causes of the dissemination of the plant and its effects and to produce information that could be used to better administer the situation. We initiated our studies considering the existence of variations in the perceptions of individual community members concerning the socio-ecological impacts of the dispersal of *A. mangium* onto indigenous lands. As such, we examined various human aspects (gender, age, leadership functions, and the time of residency in the community), assuming that certain roles within the indigenous communities would favor greater or lesser contact with this invasive species and therefore influence the acquisition of knowledge concerning it.

The records presented here of indigenous local ecological knowledge concerning the invasion of the Moskow and Malacacheta communities by *A. mangium* were based on informant responses to the following questions: (1) what are the principal problems that the Wapichana and Macuxi communities attribute to the invasion by acacias? (2) What are the uses/importance of these trees to these indigenous populations? (3) Are there relationships between perceived problems and human factors, such as gender, age, leadership roles, and residence time? (4) How do the indigenous communities explain the dispersal of the acacias in terms of their LEK?

## Methods

### Study areas

**Fig. 1:**
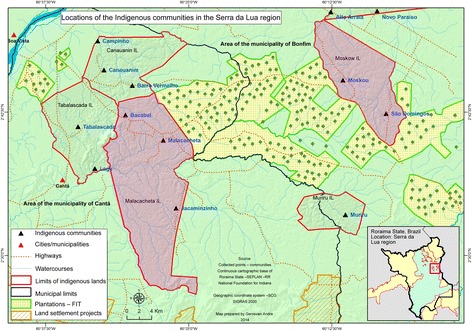
Map of the study area, showing the Malacacheta, Moskow, and São Domingos indigenous communities in the Serra da Lua Region, Roraima, and the commercial acacia plantations in the vicinity of the indigenous lands

The Moskow Indigenous Lands cover 14,212 ha in the municipality of Bonfim, Roraima State, Brazil, near the border of the Cooperative Republic of *Guyana*, and 67 km from the city of Boa Vista [21]; the Moskow community is located at 02°45′17” N; 60°11′15” W. Nearby is the São Domingos community (02°41′45” N; 60°07′28” W). The Malacacheta Indigenous Lands cover 28,631 ha in the municipality of Cantá, 37 km southeast of Boa Vista; the Malacacheta community is located at 02°40′09” N; 60°27′25” W [22] (Fig. 1). According to a census undertaken by community health workers in 2015, 105 families (approximately 450 individuals) live in Moskow, 37 families (approximately 173 individuals) live in São Domingos, and 220 families (approximately 1060 individuals) live in Malacacheta.

The present study was developed to focus on these three communities based on their geographic location and the interest expressed by their leaders in being included within the “Social Mapping of the Social-Environmental Impacts of *A. mangium* on the Indigenous Lands in Roraima” project. The mapping was undertaken by the New Amazonian Social Cartography Project, Roraima Nucleus, of which the first author was a participant. During the course of project activities, various complaints and problems related to invasive species were raised by the members of 16 indigenous communities in four municipalities in three regions of Roraima. Among these regions, the Serra da Lua stood out as having the greatest number of communities impacted by *A. mangium* [23], where that species is considered a non-native invasive plant that was previously unknown to the indigenous communities there until it was commercially planted in areas contiguous to their lands.

### Ethical and legal aspects of the research

As this research project involved access to traditional knowledge associated with biodiversity, approval was first sought from the Ethics Committee on Research with Humans at the Federal University of Amazonas and from the National Committee of Ethics in Research (n° 1.178.112/2015); authorization was subsequently granted by the National Institute of Artistic and Historical Heritage (n° 11/2015DPI/IPHAN) and the National Indian Foundation (n° 113/AAEP/PRES/2015). The research proposal itself was presented in community meetings that involved the participation of resident adult men and women, as well as young people and children; the objectives and methods to be used in this study were explained, and their approval and participation in the study was requested. During these meetings, we obtained the signatures of local community leaders on Release Forms and the willing adhesion of the community members to the research project. During the individual interviews, Free Consent and Understanding Terms (TCLE in the Brazilian acronym) were signed by all informants; individuals not able to sign their own names were asked to apply their fingerprints to the documents. These steps follow the recommendations of the National Health Council—National Commission of Research Ethics (Resolution n° 196/96).

### Information concerning the informants

Informant selection was performed based on data collected from local health centers and included community leaders and ex-leaders, foremen, teachers, and other community members. We also selected informants during social mapping activities. A total of 94 people participated in the project as informants (Table 1), 90 from the Wapichana ethnic group and four from the Macuxi ethnic group, of both sexes, with ages between 18 and 76 (average 45 ± 14.4), with an average time of residence in their respective communities of 21 ± 16.4 years.

**Table 1:** Numbers of men, women, leaders, and non-leaders, interviewed from the Moskow, São Domingos, and Malacacheta communities, in the Serra da Lua region, Roraima State, Brazil

Communities	N° interviewees	Men	Women	Leaders	
				Yes	No
Moskow	34	16	18	12	22
São Domingos	20	13	7	5	15
Malacacheta	40	24	16	8	32
Total	94	53	41	25	69

### Social-cultural dynamics of the Wapichana and Macuxi indigenous communities

The Wapichana and Macuxi are members of distinct linguistic families that have long shared the same political and geographic space [21]. The Wapichana belong to the Arawak linguistic family and inhabit savanna and forest regions in Roraima State, Brazil, as well as the Rupununi district in the Cooperative Republic of *Guyana* [24]. The Macuxi belong to the Carib linguistic family and inhabit the frontier region between Brazil and Guyana; their territory in Brazil is composed of three blocks: the Raposa Serra do Sol Indigenous Land, which contains the greatest part of their population; the São Marcos IL; and small areas in the Uraricoera, Amajari, and Cauamé river valleys [25]. Migrations and inter-ethnic marriages have favored the migration of the Macuxi to the Serra da Lua region [26], where the Malacacheta and Moskow ILs are located.

The social-cultural organizations of their communities are based on collective activities; one example is the social nature of agricultural activities, in which all family members participate in farming chores. There are important divisions of labor between family members, however, with the men dealing with chores that require greater physical effort (e.g., cutting down and chopping up trees to be burned to clear agricultural plots), while the women, in addition to taking care of their children and performing routine domestic activities (e.g., cooking and cleaning), are responsible for planting and maintaining the crops (accompanying crop development, weeding, and harvesting). The men also hunt and fish to provide for additional family needs [26].

### Data collection

During the study, indigenous knowledge concerning the invasion of acacia trees was investigated through semi-structured interviews (Appendix). These encounters were undertaken in localities designated by local leaders (generally school buildings, the central community meeting building [“*malocão*”], or in the houses of interviewees). When an informant spoke only his/her native language, translators were used. Another research technique employed was direct observation during visits to farm plots; these visits were undertaken during the acacia mapping phase of the project and during our participation in events undertaken in the different communities (which presented opportunities for collecting additional information concerning the social-cultural dynamics of those communities). Open-ended questions were used to investigate the appearance, development, and management of acacia plants, allowing flexibility in the interviewees’ responses and generating opportunities to explore other subjects pertinent to the research project, as suggested by Albuquerque [27]. The interview routine included two sections of questions exploring: (1) ethno-knowledge of the invasion process (environmental alterations and plant dispersal in the ILs); and (2) the importance and uses of acacia plants.

During the information collection phase of our study, visual stimulation was found to be a very important resource, using images of plant species in different lifecycle phases that were presented together with questions concerning the recognition of these species and the localities where they could be encountered within the ILs. Direct field observations were also employed during numerous visits to the indigenous communities between 2013 and 2015. These contacts also favored the establishment of social relationships between researchers and informants, and occurred during the data collection phase when we visited different environments in and around the communities, during community meetings and diverse social manifestations, as well as during the organization and realization of community events.

Information recorded in workshops and mini-courses, as well as other activities linked to the social mapping of the impacts of acacia plants available in the databanks of the New Social Cartography of the Amazon Region (PNCSA), Roraima Nucleus, were also accessed because of the link between the present study and the PNCSA-Roraima Nucleus beginning in 2012 (when the activities of social mapping first began). On these occasions, cognitive maps were used to discuss and represent the social-environmental problems faced by the indigenous communities after *A. mangium* plantations were established in the region. The data published here therefore includes information collected during the development of PNCSA activities, as well as that collected during our field work.

### Data analysis

The information collected during the semi-structured interviews was systematized, and the frequencies of the responses were calculated as percentages (based on the total of 94 interviewees). In order to exemplify indigenous knowledge concerning invasive plants and demonstrate research results, we selected appropriate quotes from the interviews and reproduced them here. For ethical reasons, and in agreement with the TCLEs, we have not identified the interviewees.

The present study considered variables with scalar characteristics (such as age, numbers of problems, and years of residency), nominal characteristics (such as gender or if an individual has a leadership role in the community), as well as perceptions of alterations of daily routines and the nature of those alterations. We used Pearson’s chi-square test (χ^2^) to confirm associations between pairs of nominal variables: gender x perceptions of alterations in routines; leadership roles x perceptions of alterations in routines; gender x types of alterations; leaders and nonleaders x types of alterations. We used the Mann–Whitney test (Z) to determine if there were differences between scalar variables and nominal variables with only two categories: numbers of problems x leadership roles; numbers of problems x gender; and years of residence x changes in daily routines. Finally, we used Spearman’s coefficient (ρ) to relate variables with scalar characteristics: age x numbers of environmental alterations [28]. The analyses were made using SPSS version 23.0 software. A 5% significance level was adopted for all statistical tests [29].

## Results

### The principal problems identified by the informants

The context of invasions by acacia trees is described by a community leader in São Domingos during field research activities related to the social mapping of PNCSA in 2012:

*We live on an island surrounded by acacia plants! Before, we hunted and fished, now we have bees that attack us and acacia plants that invade our farm plots as soon as we clear (burn) them, and they grow even stronger. I’ve killed rattlesnakes there that are attracted by the rats, and there have been more foxes and opossums, which damage the buriti palms. There are no more electric eels, and the water is rusty. You can’t drink the water in the Manoá igarapé, and even our wells are drying up. The ingá trees have stopped producing fruit since the acacia appeared. Parrots used to make nests in São Domingo, but now the bees have taken over. Rolinha doves used to wake us up and tell us when it was going to rain; now those birds don’t exist here anymore.*

Among the community members consulted during the research phase, most (89%) expressed their perception of changes in IL community routines after invasion by *A. mangium*. Only 11% of the interviewees did not identify the acacia plants as causing any problems. For most of the informants, the invasive plants resulted in scarcities of essential natural resources for the community and caused numerous social-environmental problems. The interviewees cited an average of two problems (varying from zero problems cited to a maximum of five). The most frequent citations referred to the degradation of water resources (71.3%), farming losses (60.6%), increased labor necessary to prepare and maintain their crop fields (41.5%), and disturbances of the local fauna (52.1%—principally caused by increases in bee populations that impede access to natural resources through their threatened or real attacks) (Table 2).

**Table 2:** Problems caused by the invasion of *A. mangium* that has altered the routines of the indigenous residents of the Moskow and São Domingos communities in the Moskow and Malacacheta Indigenous Lands, in the Serra da Lua region, Roraima State, Brazil

Problems cited	Numbers of citations	% of interviewees
Alterations of water resources	67	71.3
Prejudicial to cultivation	57	60.6
Disequilibrium of the fauna	49	52.1
Well and river water drying more rapidly	44	46.8
Altered water color	44	46.8
Increased presence of bees	39	41.5
Increased work for farm plot preparation	39	41.5
Restricted access to surrounding lands	22	23.4
No problems with the water	14	14.9
No problems cited	10	10.6
Increased presence of snakes	8	8.5
Increased presence of rats	7	7.4
Well water salty	3	3.2

Both direct and indirect problems were cited in relation to water resources: diminishing water volumes (in wetlands, rivers, and wells); alterations in water color (turbidity); well water becoming salty; and wells having reduced volumes or even drying up (Table 2). Among the arguments used by indigenous interviewees to support their negative evaluations of acacia plants, the most important was the fact that they “suck” the water from rivers and wells that serve their communities. This factor was cited as the most important effect of those plants on the environment—especially as the interviewees associated the low water levels in the rivers with their proximity to commercial plantations of acacia; in their eyes, acacia trees have deep roots and remove large quantities of water from the soil. There were likewise common complaints that acacias were making the water “*bad*,” “*dirty*,” “*red*,” or “*rust-colored*,” degrading its quality for community consumption. These indigenous populations fear that the situation will become much worse in the future, for it has become increasingly difficult to draw water from their wells: “*now we have to dig deeper wells to find water.*” These reports of changes in water quality and availability make investigations of current environmental risks imperative, especially in relation to threats to water resources that are used for diverse daily activities.

In the category related to the disequilibrium of the regional fauna are problems related to the increased presence of bees, snakes, and rats and diminishing quantities of fish because of the problems with water. According to the informants, transformations affecting the natural ecosystem include the appearance of large rats, apparently seeking food resources no longer encountered in acacia plantations. Similarly, increases in the numbers of snakes have been reported due to the greater accumulation of leaf litter in commercial acacia plantations. Another serious problem attributed to the acacia is the proliferation of Africanized bees. These bees make travel to fishing spots, or areas with *buriti* palms (to collect material used in making handicrafts), and hunting much more difficult; numerous bee attacks on people and animals have been reported. Additionally, the bees are thought to drain the floral resources of cultivated plants (maize, beans, squash, and bananas) and suppress their development.

*Our food plots used to produce many fruits, like bananas and pineapples, but now nothing grows well.*

*The acacias are attracting many noxious creatures to our lands, and we don’t have much maize because of the bees, because when the maize begins to flower the bees drain them.*

*We are surrounded by acacia, but those plants don’t yield any fruits that we can eat, and those plants are creating many problems because of the bees that chase away the little jandaíra bees that gave us their honey we used to make medicines for our children, syrups, and other remedies.*

According to the interviewees, invasive plants can appear at any time in the farm plots, where they grow very rapidly and compete with the cultivated plants—making it imperative to remove them to permit the healthy development of the plot. The local residents complained that removing those invasive plants significantly increased the maintenance efforts needed for farming. This perception varied from 25% among the inhabitants of Malacacheta to 70% in Moskow and 80% in São Domingos.

There are reports from the São Domingos community of the necessity of removing acacia trees from old farm plot localities. The trees are generally burnt, but their remains are left on the ground (because removing them demands excessive effort and energy). After burning the vegetation at the beginning of the rainy season, new seedlings germinate and must be removed. The most efficient way to remove them is to pull them out by their roots; only that will guarantee that they will not re-sprout. If the seedlings are allowed to grow they will be more difficult to remove in the future and will be prejudicial to the cultivated plants because their roots take up a great deal of the soil moisture and leave it too dry for root crops (such as sweet potato and sweet and bitter manioc) and they will not develop well. The following statements are illustrative of this situation:

*The acacias are the first things to germinate in farm plots, they germinate real pretty, better than the manioc. If I could live off the acacia I wouldn’t even need to plant anything. The acacias stunt the manioc plants, which stay small, tiny, and the roots also stay small.*

*I weed out the acacia first thing because they form many roots quickly, and if you just cut them they’ll grow right back. You can’t let them grow, or they’ll produce more seeds.*

Abandoned farm plots (“*capoeira*” or fallow) are frequently visited to collect fruits and roots (pineapples, bananas, papaya, sweet potatoes, yams, and manioc stems for planting), but they are increasingly dominated by the rapidly proliferating acacia trees. This is extremely vexing, as the farmers will have to remove the trees to clean the land when the plots are replanted (after lying fallow). There are reports of people giving up planting a plot due to the excessive work required to remove all of the acacia trees, as explained by a resident of São Domingos: *“I just didn’t want to plant there again, I was going to just use a machine to clear the area, but the acacias are very nasty plants.”*

Approximately 49% of the interviewees stated that acacia plants are very different from the native species they are familiar with. They noted significant differences in the development of the acacias, and likewise attributed impacts to the cultivated garden plants to these invaders:

*The acacias grow more rapidly than garden plants, they ruin the garden plots, after three months you have to rip the acacias out again.*

*Acacias grow very fast. Where that fellow M. had his farm plot in São Domingos is pure acacia now.*

*Acacia grows green and pretty, and the leaves don’t fall.*

*Acacia grows more rapidly than manioc or the other plants cultivated in our gardens. After it flowers, lots of seeds are produced, they germinate and grow even in the dry season, with nice green leaves; the leaves don’t fall, and the leaves are different – long and thick when it starts growing and then they change.*

Descriptions of the problems caused by acacias were reinforced by the statements of a community leader (“*tuxaua*”) from the Moskow IL, who has a large family and a large farm plot with manioc, squash, watermelons, beans, maize, sweet potatoes, papaya, green peppers, taioba, and peanuts) 13 km from a commercial acacia grove. He stated that:

*The farm plot used to grow really nice bitter manioc, beautiful sweet manioc, now it’s pure acacia. The rivers had lots of fish in those days and we caught lots of them. Now we have acacia damaging our plants. Our community here is being seriously hurt by the acacias.*

Many of the indigenous residents manifested their discontent with the restrictions being imposed on movements within their own lands. In some places, they now have to ask permission from the owners of the commercial acacia plantations to go through them; quite frequently they are stopped at the entrances to the plantations to explain where they are going and why.

### The uses and importance of acacias

On being asked about the uses and importance of acacias, most of the informants (89%) stated that they could think of no uses at all for these invasive plants. For these people, acacias had no positive importance for the communities, and they categorically stated that they had no future expectation of ever being able to use the acacias for any beneficial purpose: “*acacia wood isn’t good for anything, it’s too soft.*” As an informant from São Domingos noted: “*to me they are just a pest that has fallen upon our community.*” There are some reports of uses for these invasive trees as firewood for drying manioc flour or for supplying fibers (“*envira*”) for binding wood and manioc; it is also said that the trees are useful for their copious shade. These positive citations are only occasional, however, and the useful practices cited are apparently quite sporadic.

Sentiments of revulsion and fear were quite perceptible among the interviewees in terms of the futures of their communities. In general, *A. mangium* was viewed as a pest species that did not, and never would, bring any benefits at all to the community and would never become an important product in the local economy—a plant that would only generate difficulties, such as those described in relation to farming practices and decreasing productivity, with grave consequences for community subsistence. Some interviewees, however, considered that they lacked more information about the possibility of acacias generating benefits for their community.

### Gender and leadership

The invasive *A. mangium* has altered the routines of the indigenous communities in Roraima, generating problems that affect their subsistence. As such, acacias were considered prejudicial by 89% of the members of the Malacacheta, Moskow, and São Domingos indigenous communities. A more detailed analysis of the information gathered indicated the lack of significant differences between the opinions of men and women (χ^2^ = 0.99; *p* = 0.753) and leaders and nonleaders (χ^2^ = 0.180; *p* = 0.671) in terms of their perceptions of alterations in their routines attributable to the occurrence of acacia plants in the ILs. Similarly, there were no significant differences in terms of the types of environmental impacts cited by men or women (χ^2^ = 5; *p* = 0.691) or by leaders and nonleaders (χ^2^ = 5; *p* = 0.46).

The Mann–Whitney test, however, indicated that the local community leaders (*tuxauas*) could more precisely describe the types of problems encountered than could nonleaders (Z = − 0.2078; *p* = 0.038). The same test did not demonstrate significant differences between men and women in terms of the numbers of environmental alterations cited (Z = − 0.217; *p* = 0.828). It is important to note that the community leaders tended to report all of the problems that occurred in the communities. To be a community leader means occupying the highest position in the social hierarchy of an ethnic group, a person who represents their community in all local and external events, and who must always conduct themselves in a manner that guarantees the well-being of their people. The leaders had sought the support of the PNCSA-Roraima Nucleus to publicly express their perceptions and concerns about the propagation of acacias throughout the ILs based on collective opinions and decisions.

### Age and residence time in the community

There was a positive correlation between age and the numbers of citations contributed by an informant (rho = − 0.275; *p* = 0.007), indicating that older people mentioned more types of environmental alterations attributable to invasive plants. Individuals that had lived for longer periods of time in the communities were not only the most emphatic in their affirmations that there were palpable alterations in the routines of the community members due to the invasion by acacia trees, but they also provided more supporting information (Z = − 1.965; *p* = 0.049). This type of knowledge can be seen in a section of a statement provided by one of the leaders who helped found the São Domingos community and lived on the IL before there were acacia plantations:

*[…] it wasn’t like that before, after burning for clearing now, only acacia seedlings germinate, and when you’re ready to plant – there she is again, the seedlings germinate before the grasses and grow rapidly; and the rains help, acacias really like water […].*

### Local ecological knowledge concerning acacia dispersal in the ILs

In terms of the LEK of the indigenous populations concerning the dispersal of acacias within the Moskow and Malacacheta ILs, 37% of the interviewees stated that their dispersal was intermediated by birds, by wind (10%), or by bats (6%). However, 46% of the interviewees stated that they had no explanation for the rapid dissemination of those invasive plants to localities distant from the commercial plots. The following statement expressed their views on dispersal:

*[…] There is an area in the Malacacheta reserve that has only acacias, they also grow near the neighbor’s house, even 4 or 5 km in from the border of Malacacheta there are acacia plants, so you ask yourself, how did those acacias get there? Their seeds are very light, and the wind and birds will take them, and wherever they fall an acacia will germinate […].*

According to the interviewees, burning the vegetation (67%) and rainfall (70%) intensified germination—creating concern among community members in relation to the rapid advance of acacias in secondary vegetation and invading fallow plots (“*roça velha”)*. During the interviews, many of the interviewees expressed in their answers worries and lamentations about *A. mangium*:

*What is our future going to be like? I already know, the acacias suck all the water from the soil and leave it really dry, I have to pull the maniac roots from the hard clay, it’s tiring work.*

*My fear is that in the future we’re going to be without any water if we can’t control the acacias. Who’s going to cut the acacia trees in the future, my sons?*

*We’re going to have a lot more trouble in the future because of the acacias. I’m worried about the fact that they could take over everything, because they’re already just about everywhere: the forest, our farm plots, the banks of the rivers – I’m worried that if in the future our children have any way to live, everything will just turn into acacia, my worry is that they will do away with the native plants.*

Some of the indigenous residents felt that the acacias were unknown plants, as they did not know where they came from or what they were used for—even though there are so many commercial stands of acacia. It became very clear from the interviews that the local inhabitants knew very little about the commercial uses of acacia trees and even less about their benefits— “*the trees that do not produce fruits*” that could be seen rapidly changing the lives of the people in the surrounding communities.

## Discussion

### Principal problems identified by the informants

In our study of the situations being experienced by the Wapichana and Macuxi people, it became very clear from their statements that environmental changes due to a decade of interactions with the non-native species *A. mangium* were seriously affecting their normal routines. One of the most frequent observations of the interviewees referred to the drying of rivers, lakes, and community wells—which are associated, according to the informants, with the abundant propagation of those invasive plants and their insatiable thirst for water. This observation has generated a good deal of concern among the indigenous populations in terms of the economic, ecological, cultural, and social sustainability of their communities. The absence of up-to-date scientific data concerning the true water consumption rate of *A. mangium* does not permit a better grasp of the subject, although it is important to note that the black acacia (*A. mearnsii*), a species that has invaded a wide variety of vegetation types in South Africa, has been shown to use substantially more water than the native vegetation [30]; equally, it is known that evapotranspiration from humid area vegetation is greater than that of dryland vegetation. It would therefore be reasonable to assume, within the context of the communities in Roraima State, that the dense growth of *A. mangium* trees in commercial plantations near natural watercourses constitutes a significant drain on groundwater resources, because of the lack of balance between plant transpiration and groundwater replacement through rainfall [3].

Similarly, the indigenous populations of Roraima State have noted the high germination rates of acacia seedlings after burning the cover vegetation (the first step in preparing the land for farming) and at the beginning of the rainy season, as have other authors in studies of invasive plants [6, 15]. According to Le Maitre et al. [6], Australian acacias commonly accumulate persistent soil seed banks that allow them to rapidly occupy and dominate disturbed sites. The seed bank of *A. mangium* favors subsequent invasions, that is, the growth of new acacia seedlings following the indigenous practice of preparing the land for planting by cutting the cover vegetation and then burning everything. The Soliga ethnic group likewise recognized the influence of fire on stimulating the germination of the invasive species *Lantana camara* [2]. It is quite probable that the soil management techniques used by the indigenous populations studied here, including slash and burn agricultural methods, are favoring the propagation of acacias in their farm plots. The informants indicated that acacias inhibit the full development of manioc and other cultivated plants and dominate fallow fields (*roça velha*). Similar observations, although in forest environments, were described by Sundaram et al. [2], who reported impacts on the structures of native vegetation by the invasive species *Lantana camara*, as well is the positive influence of fire on its propagation. After invasion by *L. camara*, it becomes more difficult to harvest tubers in farm plots [2]. These authors also concluded that the observations of indigenous residents aided in a better understanding of the invasion process and would assist in the management and control of that aggressive species [2].

According to the LEK recorded in the social mapping efforts of the New Social Amazonian Cartography Project (2014), *A. mangium* generates social-environmental impacts due to its wide dissemination in different IL habitats. It must be recognized, however, that this subject has not yet been fully investigated, and a detailed scientific study would be needed to confirm the dispersal capacities of *A. mangium* propagules [31]. Those authors identified individuals that had dispersed into natural savanna ecosystems located more than 900 m from commercial plantations [31]. According to local informants, invasive acacias can be readily found in *buriti* palm stands, near wells, along river banks, and in new and abandoned farm plots.

Negative impacts on the regional fauna were cited by the indigenous informants as important environmental side effects of economic development and human occupation. The transformation of the natural landscapes near the ILs due to monoculture acacia plantations, the installation of cattle farms, deforestation, and burning have all contributed to upsetting the equilibrium of the fauna near the indigenous communities of Serra da Lua [22]. The marked increase in bee populations following the spread of acacia plants has created great consternation among the communities there due to numerous attacks on humans and animals; additionally, it has been noted that some cultivated plants visited by Africanized bees do not develop as well as they did before. These observations agree with the results of a study by Oliveira and Cunha [19], who found that Africanized bees are more common in open areas, where they can easily harvest resources from pioneer and invasive plants. This disequilibrium of the local fauna was actively discussed during the training activities of local community members to become environmental agents within the Permanent Vigilance, Management, and Territorial Training Program initiated by the Indigenous Council of Roraima [22].

The informants in the IL stated that the dissemination of *A. mangium* resulted in the undesirable ecological consequences mentioned above. Hence, these situations will require direct mitigating actions by the company responsible for the commercial plantations—by working together with the indigenous communities who have intimate ecological knowledge of their lands.

### Dispersal of *A. mangium*

The LEK of the São Domingos indigenous community is not precise about the dispersal mode of acacia seeds to sites distant from their commercial plots of origin, but birds, bats, and wind are mentioned as probable dispersal agents. These observations are consistent with Kull and Rangan [32], who determined that birds, together with the wind, are the principal dispersal agents of *A. mangium* seeds. As such, the inclusion of indigenous ecological knowledge concerning the invasive species *A. mangium* into management projects, together with the participation of these ethnic groups in monitoring invaded areas, will be indispensable for controlling these plants.

The experiences of indigenous populations with invasive plants have allowed them to accumulate important information concerning these species that is in agreement with the scientific information available in the literature and has alerted them to some of the multiple facets of its invasive processes, including its manner of dispersal. This local knowledge is important and should be recognized as useful when formulating plans for non-native species management.

### Importance and use of acacias

Interactions between indigenous groups and acacia plants have been occurring for more than two decades now, as could be confirmed by the immediate recognition of this invasive species in illustrations shown to the interviewees. The plant is thought of as a tree that does not produce any fruits and has very soft wood that renders it essentially useless for constructing traditional residences, etc. Local perceptions of this plant are thus quite negative, and the indigenous populations cannot envision any utility for it, or future benefit from it, in terms of their traditional lifestyles. When this subject was raised during community assemblies, *A. mangium* was the target of severe criticisms by local inhabitants, who were discontented with the environmental changes associated with its appearance. In other communities, however, Australian acacias generate income for rural populations in spite of their invasive behavior and some negative effects on traditional lifestyles [7, 8]. It is interesting that not even the high caloric content of acacia wood (~ 4900 kcal/kg—which led to the establishment of plantations for producing firewood [33] and has been noted by the indigenous individuals when burning their farm plots to clean them) has persuaded them to change their negative perceptions of this imported plant. According to some of the authors consulted during the present study [7], attitudes toward invasive species are directly related to perceptions of opportunities to use them and/or generate income from them, which can lead to changes in their attitudes of acceptance and better adaptations to the new social/economic contexts created by these plants.

In general, the populations that benefited from introduced acacias were not previously informed about the potential ecological impacts of these invasive plants on natural ecosystems, and when their local natural resources became scarce they were left to deal with these negative effects on their own [23]. Nonetheless, some invasive Australian acacias have presented important opportunities for generating income and subsistence among rural populations [7]. These non-native plants are not considered a resource that will become rare, and some communities have chosen to plant even more non-native plants in detriment to natural native species [9], without considering probable undesirable consequences, whether in terms of long-term environmental damage and/or financial losses that were not factored in. As such, alternative sources of economic benefits were evaluated only in terms their intrinsically advantageous characteristics (rapid growth, high productivity, and high abundance).

The statements of the indigenous community members interviewed are dominated by negative comments concerning *A. mangium*, many of which are mentioned in the text above. These problems have contributed to the disinterest of the local indigenous populations in commercially exploiting *A. mangium* or contemplating any practical use for it—in contrast to populations in other countries that have been able to obtain economic benefits from invasive plants [7]. Even residents who received saplings as “gifts” at the very start of the project reacted in an unexpected manner after our first interviews—by simply cutting and burning the plants that were growing in their yards.

### Indigenous knowledge in terms of gender, leadership status, age, and time of residence

Most of the statistical tests did not identify significant differences in the perceptions of invasive acacia plants by men or women within the dynamics of their community life. Community leaders, as well as older residents, were aware of larger numbers of problems associated with *A. mangium*. Informants that had lived in the indigenous communities for longer periods of time noted that there had been significant changes in local routines due to the uncontrolled propagation of acacia. Different perceptions concerning the effects of invasive plants were observed in terms of informant age and gender [10], with older residents being especially concerned with the loss of local biodiversity; the men were aware of favorable changes for farming and the positive responses of sweet potatoes to the presence of *A. mangium*, while the women noted the marked disappearance of mushrooms.

In terms of the acquisition of local knowledge [34], it is presumed that the ecological awareness of indigenous populations concerning the invasion by *A. mangium* is the result of personal observations and experiences within a new ecological and social environment, combined with personal experiences attempting to minimize the impacts of acacias. In this sense, local knowledge comes from experience with the processes of invasion and can generate insights concerning the restoration of damaged environments. Le Maitre et al. [6] recognized that both social and ecological considerations are important for the successful recuperation of impacted ecosystems.

It was noted that indigenous men and women, both young and older, had the same unfavorable opinion concerning the invasive plants as did community leaders and could enumerate large numbers of negative consequences related to the appearance of *A. mangium*. Their positions reflect, in part, the lack of precise information provided to them when the acacia plantations were first being organized as, according to the informants, the possible negative consequences of the introduction of this non-native species near the IL were not clearly and openly explained [35].

## Conclusions

The majority of the informants interviewed in the three communities believe that the acacia plants have negative effects on the natural environment and on the subsistence of their communities, because of the many different problems that arose with their uncontrolled propagation. The observed impacts of the acacias (and the lack of any attempts to use them) made any possible utilitarian value seem absolutely insignificant. The similarities between the opinions of men, women, and community leaders show that the knowledge concerning the acacias is well distributed through sociocultural relationships.

It is important that this indigenous ecological knowledge be included in future evaluations of *A. mangium*, in management plans designed to contain its dispersal beyond the limits of the commercial plantations, and to mitigate its impacts on local socio-ecological systems. As such, we recommend that all new commercial plantations be established at secure distances from indigenous agricultural plots (based on local experiences with *A. mangium*) to minimize their negative impacts on local ecosystems and to control the species’ invasive propensity.

## References

[1] Pysek P, Richardson DM (2010). Invasive species, environmental change and management, and ecosystem health. Annu Rev Environ Resour.

[2] Sundaram B, Krishnan S, Hiremath AJ, Joseph G (2012). Ecology and impacts of the invasive species, *Lantana camara*, in a social-ecological system in South India: perspectives from local knowledge. Hum Ecol.

[3] Projeto Nova Cartografia Social da Amazônia—PNCSA. Boletim Informativo Mapeamento social como instrumento de gestão territorial contra o desmatamento e devastação: defesa dos territórios tradicionais, n. 5. Manaus: UEA Edições; 2014.

[4] Projeto Nova Cartografia Social da Amazônia—PNCSA. Fascículo Mapeamento social como instrumento de gestão territorial contra o desmatamento e devastação: invasão da *Acacia mangium* nas terras indígenas de Roraima, n. 15. Manaus: UEA Edições; 2014.

[5] Pimentel D, editor. Biological invasions: economic and environmental costs of alien plant, animal, and microbe species. Boca Raton: CRC Press; 2014. p 384.

[6] Le Maitre DC, Gaertner M, Marchante E, Marchante E, Ens EJ, Homes PM, Pauchard A, Ofarrell PJ, Rogers AM, Blanchard R, Blignaut J, Richardson DM (2011). Impacts of invasive Australian acacias: implications for management and restoration. Divers Distrib.

[7] Kull CA, Shackleton CM, Cunningham PJ, Ducatillon C, Dufour-Dror JM, Esler KJ, Friday JB, Gouveia AC, Griffin AR, Marchante E, Stephen JM, Pauchard AP, Ragan H, Richardson DM, Rinaudo T, Tassin J, Urgerson LS, Von Maltitz GP, Zenni RD, Zylstra MJ (2011). Adoption, use and perception of Australian acacias around the world. Divers Distrib.

[8] Santos LL, Nascimento ALB, Vieira FJ, Silva VA, Voeks R, Albuquerque UP (2014). The cultural value of invasive species: a case study from semi–arid northeastern Brazil. Econ Bot.

[9] Shackleton CM, Mcgarry D, Fourie S, Gambiza J, Shackleton E, Fabricius C (2007). Assessing the effects of invasive alien species on rural livelihoods: case examples and a framework from South Africa. Hum Ecol.

[10] Siges TH, Hartemink AE, Hebinck P, Allen BJ (2012). The invasive shrub *Piper aduncum* and rural livelihoods in the Finschhafen area of Papua New Guinea. Hum Ecol.

[11] Richardson DM, Pysek P, Rejmánek M, Barbour MG, Panetta FD, WEST CJ (2000). Naturalization and invasion of alien plants: concepts and definitions. Divers Distrib.

[12] Berkes FCJ, Folke C (1998). Rediscovery of traditional ecological knowledge as adaptive management. Ecol Appl.

[13] Marioti PS, Santos JE, Pires JSR (1998). Caracterização perceptiva de uma área natural de conservação por docentes do ensino fundamental. Rev Univille.

[14] Mwangia EB, Swallowb B (2008). Prosopis juliflora invasion e modos de vida rurais na lake baringo área do Quênia. Conserv Soc.

[15] Delnatte C, Meyer JY (2012). Plant introduction, naturalization, and invasion in French Guiana (South America). Biol Invasions.

[16] Rossi LMB, Azevedo CP, Souza CR. *Acacia mangium*. Manaus: Embrapa/Am Ocidental; 2003. 29p.

[17] Matthews S, Brand K (2006). América do Sul invadida: a crescente ameaça das espécies exóticas invasoras.

[18] Barbosa RI, Campos C, Pinto F, Fearnside PM (2007). The “lavrados” of Roraima: biodiversity and conservation of Brazil’s Amazonian savannas. Funct Ecosyst Commun.

[19] Oliveira ML, Cunha JA (2005). Abelhas Africanizadas *Apis Mellifera scutellata* lepelletier, 1836 (*Hypnoptera*, *Apidae* e *Apinae*) exploram recursos na floresta amazônica?. Acta Amaz.

[20] Rai RK, Rai R (2013). Assessing the temporal variation in the perceived effects of Iivasive plant species on rural livelihoods: a case of Mikania micrantha invasion in Nepal. Conserv Sci.

[21] Miller RP, Uguen K, Pedri MA, Creado ESJ, Martins LL, Trancoso R (2008). Levantamento etnoambiental das terras indígenas do complexo Macuxi-Wapichana: Anaro, Barata, Livramento, Boqueirão, Raimundão, Jacamin, Moskow, Muriru, Tabalascada e Raposa Serra do Sol.

[22] CONSELHO INDÍGENA DE RORAIMA-CIR. Amazad Pana’Adinhan: percepções das comunidades indígenas sobre as mudanças climáticas. região Serra da Lua-RR. Boa Vista: CIR; 2014. 154p.

[23] Souza AO, Chaves MPSR, Barbosa RI, Frank N, Clement RC (2015). Uso sustentável dos ecossistemas naturais? cultivo de *Acacia mangium* Willd. nos “lavrados” de Roraima.

[24] Oliveira AR. Tempos dos Netos. Abundância e escassez nas redes de discursos ecológicos entre os Wapichana na fronteira Brasil-Guiana (Tese de doutorado). Brasília; 2012.

[25] Instituto Socioambiental (2011). Diversidade Socioambiental de Roraima: subsídios para debater o futuro sustentável da região: Org. Ciro Campos.

[26] Mandulão G (2012). Projeto vidas paralelas indígena: revelando os povos Macuxi e Wapixana de Roraima.

[27] Albuquerque U, Lucena R, Alencar N, Albuquerque UP, Lucena RFP, Cunha LVFC (2010). Métodos e técnicas para coleta de dados etnobiológicos. Métodos e Técnicas na Pesquisa Etnobiológica e Etnoecológica.

[28] Siegel S, Castellan Jr. N J. Estatística não-paramétrica para ciências do comportamento. 2ª ed. Porto Alegre: Artmed; 2006. 448p.

[29] Pestana MH, Gageiro JN (2000). Análise de dados para Ciências Sociais—a complementariedade do SPSS.

[30] Dye P, Jarmin C (2004). Implications for the link between removal of invading trees and catchment streamflow response. S Afr J Sci.

[31] Aguiar A, Barbosa RI, Barbosa JBF, Mourão M (2014). Invasion of *Acacia mangium* in Amazonian savannahs following planting for forestry. Plant Ecol Divers.

[32] Kull CA, Rangan H (2008). Acacia exchanges: wattles, thorn trees, and the study of plant movements. Geoforum.

[33] Lorenzi H, Souza HM, Torres, M, Bacher L. Árvores exóticas no Brasil: madeireiras, ornamentais e aromáticas. Instituto Plantarum; 2003. 368p.

[34] Soldati GT, Albuquerque UP (2014). A Transmissão do conhecimento local ou tradicional e o uso dos recursos naturais. Introdução à Etnobiologia. Org.

[35] Lauriola E, Barbosa, RI, Nascimento-Filho HR. Nota preliminar sobre impactos das plantações de *Acácia mangium* Willd. sobre terras e populações indígenas de Roraima. Relatório Técnico, INPA/RR; 2002.

